# Reconstruction of long digital extensor tendon by cranial tibial muscle fascia graft in a dog

**Published:** 2016-09-15

**Authors:** Soroush Sabiza, Ahmad Khajeh, Hadi Naddaf

**Affiliations:** Department of Clinical Science, Faculty of Veterinary Medicine, Shahid Chamran University of Ahvaz, Ahvaz, Iran.

**Keywords:** Dog, Fascia, Graft, Rupture, Tendon

## Abstract

Tendon rupture in dogs is generally the result of a direct trauma. This report described the use of adjacent muscle autogenic fascial graft for reconstruction of distal rupture of long digital extensor tendon in a dog. A two-year-old male mix breed dog, was presented with a non-weight bearing lameness of the right hind limb and a deep rupture of lateral side of right tarsus. History taking revealed that this rupture appeared without any apparent cause, when walking around the farm, three days before. Radiography was done and no fracture was observed. Hyperextension of right tarsal joint compared to left limb was observed. Under general anesthesia, after dissections of the ruptured area, complete rupture of long digital extensor tendon was revealed. Then, we attempted to locate the edge of the tendon, however, the tendon length was shortened approximately 1 cm. Hence, a strip of 1 cm length from fascia of cranial tibial muscle was harvested to fill the defect. The graft was sutured to the two ends of tendon using locking loop pattern. Subcutaneous layers and the skin were sutured routinely. Ehmer sling bandage was applied to prevent weight bearing on the surgical region. Re-examination and phone contact with the owner eight weeks and six months postoperatively revealed a poor lameness and excellent function of the dog, respectively. It could be concluded that the fascia of adjacent muscles can be used as an autogenic graft for reconstruction of some tendon ruptures.

## Introduction

Tendon rupture in dogs is generally the result of a direct trauma.^1 ^ The complete rupture of the tendon with tissue loss requires correct evaluation by the surgeon because of the impossibility of approaching tendon ends with a simple suture.^2^ Several studies suggested fascia lata autograft and anchor suture fixation for tendon implantation in complete rupture of the tendon.^3^^-^^5^ The long digital extensor muscle is a spindle shaped muscle that originates proximally by means of a tendon from the extensor fossa of the lateral femoral condyle and more distally, at the level of the metatarsal bones. It becomes a tendon that divides into four portions that pass along the dorsal aspect of the metatarsal and phalangeal bones of the second to the fifth digit to the end on the distal phalanx of the supporting digits.^6^^,^^7^ The rupture of this tendon in its distal end is unusual as it is not documented yet or we could not find elsewhere. Also, the use of fascia except fascia lata is not used routinely for treatment of tendon rupture. This report described the use of autogenic fascial graft for reconstruction of distal tendon of long digital extensor.

## Case Description

**Fig. 1:**
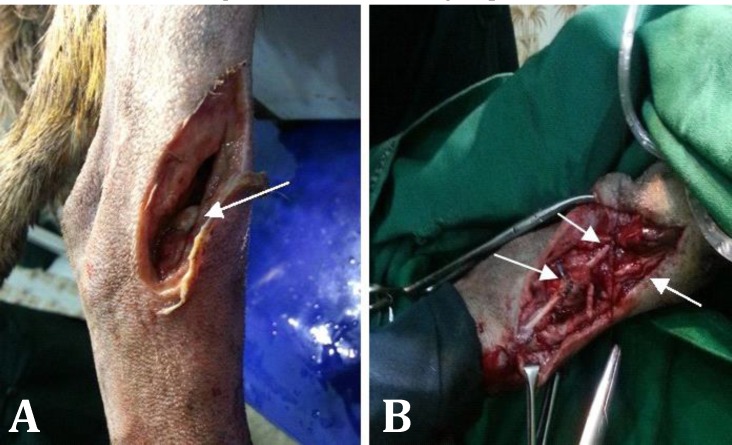
A) Deep rupture of tarsal region. The arrow shows distal end of ruptured long digital extensortendon. B) Right arrow shows cranialis tibialis muscle. Left arrows show graft suturing to the two ends of tendon using locking loop pattern

A two-year-old male mix dog, weighing 20 kg, was presented to the Veterinary Teaching Hospital of Shahid Chamran University of Ahvaz with a non-weight bearing lameness of the right hind limb and a deep rupture at the lateral aspect of the tarsus. Bleeding, inability to weight bearing on the traumatized limb and inability to walk, was evident. History taking revealed that this rupture appeared without any apparent cause, when walking around the farm, three days before. No signs of fracture were detected by clinical examination which confirmed with x-ray. Wrinkle of superficial digital flexor tendon and hyper-extension of right tarsal joint compared to the intact limb, was observed. Also, a small white piece and tendon like structure was seen that suggested one of the extensor tendons (Fig. 1A). Blood sampling was done to check hematology table before the anesthesia procedure and surgery. Hematocrit was very low (11%) while another parameters was approximately in normal ranges. First of all, we decided to stabilize the dog. Hence, a healthy male dog, was selected after the cross-match test to take the blood sample using sodium citrate (Merck, Darmstadt, Germany), for blood transfusion. Blood transfusion was done through an angiocatheter 20 which was fixed in right cephalic vein. The transfusion rate was 0.25 mL Kg^-1^ per hr in the first 20 min, and then increased to 5 to 10 mL kg^-1^ per hr.^8^ After stabilizing the patient, anesthesia was induced using a mixture of 0.2 mg kg^-1 ^diazepam (Caspian Tamin Pharmaceutical Co., Rasht, Iran) and 10 mg kg^-1 ^ketamine (Alfasan, Woerden, The Netherlands) through the angiocatheter.^9^ Then, intubation was applied to have a patent air way. Ruptured area was clipped and scrubbed. To prevent the hemorrhage during the operation, a tourniquet was applied under the knee. After dissections, complete rupture of distal tendon of long digital extensor (LDE) was revealed. Then, we attempted to locate the edge of the tendon, however, the tendon length was shortened approximately 1 cm due to elapsed time of three days. Therefore, a strip of 1 cm length from fascia of cranial tibial muscle was harvestedto fill the defect area (Fig. 1B). In this way, after trimming of the graft edges, the graft was sutured to the two ends of tendon using locking loop pattern of 2/0 polyglycolic acid (PGA; Supa Medical Devices, Tehran, Iran) with the joint kept in the same angle of the opposite tarsus joint (Fig. 1B). The fascial defect was closed using simple continues pattern with PGA 2/0. Then, the area was lavaged using saline plus povidone iodine (Daru Darman Pharmaceutical Co., Tehran, Iran). Subcutaneous layers were sutured using 2/0 PGA simple continues and the skin edges were closed using 2/0 nylon suture (Supa Medical Devices) simple interrupted sutures. Ehmer sling bandage was applied to prevent weight bearing on the surgical region for eight weeks. The dog was rested for eight weeks and given intravenous cefazolin (22 mg kg^-1^; Exir Pharmaceutical Co., Borujerd, Iran) for five days to prevent infection and ketoprofen (2 mg kg^-1^; Razak Laboratories, Karaj, Iran) for three days to control pain and inflammation. On re-examination eight weeks postoperatively, a mild lameness was still present, however, no pain could be elicited. Phone contact with the owner six months postoperatively revealed excellent function of the dog and revealed only a poor lameness.

## Discussion

To the best knowledge of the authors, this article is the first report on the rupture of distal end of long digital extensor tendon and using adjacent muscle fascia as an autogenic graft to treat it. The nature of the tendon trauma influences the type of tendon injury and may result in acute rupture of the tendon with partial or complete loss of integrity of the structure, sometimes with exposure of tendon ends. In addition, weakening or rupture of the tendon structure can occur secondarily to systemic diseases (e.g. Cushing’s disease) or to iatrogenic etiologies.^2^ There are three different types of breakage, based on anatomical location and the severity of the lesion of the tendons: type 1 is a complete rupture, type 2 has three subtypes for partial rupture with a lengthened tendon and type 3 is a tendinosis or peritendinosis.^10^ The restoration of normal tendon function after injury requires reestablishment of tendon fibers and the gliding mechanism between the tendon and its surrounding structures.^11^ In most cases of tendon laceration or rupture, surgical intervention is required to direct the natural process of healing and occasionally the damage exceeds the natural regenerative ability even with existing treatment modalities.^7^ Fascia lata autograft and anchor suture fixation is used for tendon implantation in complete rupture of the tendon.^3^^,^^5^ During post-operative management, primarily in the early stages of healing, limb immobilization is essential. Movement can produce a significant space between the tendon ends, decreasing the local blood supply and increasing fibrosis, which can compromise healing and final functional outcome.^12^ The average time of maintaining the system of immobilization was 10 weeks, while the average time of recovery for normal limb functionality was approximately 20 weeks.^13^ Fascia is essentially the same kind of tissue as is tendon, but it is thinner.^14^ It consists of many parallel layers of heavy white fibrous tissue, between which are situated a few scattered cells. The fibers are arranged in thin sheets and are divided into irregular bundles by areolar tissue, which also covers the surface and in which ramified blood vessels and lymphatics are present. Fascia is an ideal material for transplantation.^14^ It is thin and is easily permeated by serum, it has great strength. Loops of fascia fastened in a vise have withstood a weight of 40 kg. without breaking, the ends of the fascia slipping from the vise first.^14^ The deep fascia is a fibrous membrane forming an intricate network which envelops and separates muscles, forms sheaths for nerves and vessels, strengthens ligaments around joints, and binds all the structures together into a firm compact mass. The deep fasciae envelop all the muscles of the body, however, have different features according to the region.^14^^,^^15^ The deep fasciae of the limbs are well-defined laminae of connective tissue with a mean thickness of 1 mm. In particular, according to our morphometric analysis, the fascia lata has a mean thickness of 944 μm and the crural fascia 924 μm, whereas the brachial fascia is thinner (700 μm).^15^ In this case, we use a strip fascia of cranial tibial muscle as a graft to fill the defect of ruptured tendon. The ruptured tendon was fully backed to its function six months postoperatively, this result was the same with the experimental study done by Weinberg who used dead fascia grafts in tendon defects.^14^ Although ultrasonography is better than clinical performance to evaluate tendon structure due to this kind of graft, however, it was not possible for us due to no consent of the owner. Routinely, fascia lata is used as a graft because of its excellent thickness, but the other fasciae are not. This case report aimed to express the possibility of using fascia from adjacent muscles as an autogenic graft for reconstruction of some tendon ruptures. It is possible to reconstruct tendons without any more surgical incisions. Further studies should be done to verify this hypothesis.
